# Cytokeratin-19 fragments in serum (CYFRA 21-1) as a marker in primary liver cancer

**DOI:** 10.1038/sj.bjc.6601026

**Published:** 2003-06-10

**Authors:** T Uenishi, S Kubo, K Hirohashi, H Tanaka, T Shuto, T Yamamoto, S Nishiguchi

**Affiliations:** 1 Department of Hepato-Biliary-Pancreatic Surgery, Osaka City University Graduate School of Medicine, 1-4-3 Asahimachi, Abeno-ku, Osaka 545-8585, Japan; 2 Department of Hepatology, Osaka City University Graduate School of Medicine, 1-4-3 Asahimachi, Abeno-ku, Osaka 545-8585, Japan

**Keywords:** CYFRA 21-1, tumour marker, primary liver carcinoma, intrahepatic cholangiocarcinoma

## Abstract

Using an electrochemiluminescence immunoassay, CYFRA 21-1 concentrations were measured in sera from 187 patients with primary liver cancer (164 with hepatocellular carcinoma (HCC) and 23 with intrahepatic cholangiocarcinoma (ICC)) and 87 patients with benign liver diseases. Concentrations of CYFRA 21-1 were significantly higher in patients with ICC (*5.0*; interquartile range 3.1–10.7 ng ml^−1^) than in those with benign liver disease (1.4; 1.0–1.9; Mann–Whitney *U*-test, *P*<0.0001) or HCC (1.7; 1.1–*2.7*; Mann–Whitney *U*-test, *P*<0.0001). Using cutoff values selected for 95% specificity in the benign group (3.0 ng ml^−1^), CYFRA 21-1 showed higher sensitivity for ICC (*87.0*%) than three commonly used markers including *α*-fetoprotein (17.4%), carcinoembryonic antigen (34.8%), and carbohydrate antigen 19-9 (*60.9*%). Serum CYFRA 21-1 increased in ICC from stages I/II to IV (Kruskal–Wallis test, *P*=0.0102). CYFRA 21-1 concentration increased with extent of local invasion, but not nodal status. Serum CYFRA 21-1 represents a useful diagnostic test for ICC that offers high sensitivity. CYFRA 21-1 reflected differences in tumour burden, suggesting applicability to staging and follow-up.

Hepatocellular carcinoma (HCC), one of the most common primary malignant tumours worldwide, is a leading cause of death (Pisani *et al*, 1999). Since chronic infection with hepatitis B or C virus (HBV or HCV) is closely related to development of HCC (Ikeda *et al*, 1993; Nishiguchi *et al*, 1995; Takano *et al*, 1995), close follow-up of patients with HBV or HCV infection has been recommended to improve early HCC detection and maximise opportunity for successful treatment (Liaw *et al*, 1986; Curley *et al*, 1995; Trevisani *et al*, 2002). While various imaging modalities can be applied to diagnosis of primary liver cancer, the main diagnostic test remains measurement of *α*-fetoprotein (AFP), the best accepted serum tumour marker for HCC, in addition to imaging (Oka *et al*, 1994; Curley *et al*, 1995; Trevisani *et al*, 2002).

Recent studies reported an increased risk of developing intrahepatic cholangiocarcinoma (ICC) in patients with cirrhosis, as is true for HCC (Sorensen *et al*, 1998; Kobayashi *et al*, 2000). In Japan and east Asia, chronic HCV infection has been linked to a high incidence of ICC, including combined hepato-cholangiocellular carcinoma (c-HCC-CC) (Tomimatsu *et al*, 1993; Shin *et al*, 1996; Su *et al*, 1996; Taguchi *et al*, 1996; Yamamoto *et al*, 1998). Follow-up of patients with HCV infection therefore can detect many cases of ICC as well as HCC. Although serum concentrations of carcinoembryonic antigen (CEA) and carbohydrate antigen (CA) 19-9 are commonly measured to detect and monitor of ICC, insufficient sensitivity and specificity has been a problem with using these established markers in this form of cancer (Kawarada and Mizumoto, 1984, 1990; Wang *et al*, 1994; Yamanaka *et al*, 1995; Nakamura *et al*, 1996; Chu *et al*, 1997; Kim *et al*, 1999). A more accurate marker for ICC is needed.

In malignant epithelial cells, activated protease increases degradation of cytokeratin; this results in release of large amounts of cytokeratin fragments into the blood (Dohmoto *et al*, 2001; Wu *et al*, 2002). The CYFRA 21-1 assay was developed to measure a soluble fragment of cytokeratin 19 in serum. In non-small-cell lung cancer, CYFRA 21-1 was found to be significantly more sensitive than established markers, and this test may be a useful adjunct in clinical monitoring during and following treatment (Pujol *et al*, 1993; Stieber *et al*, 1993; Sugama *et al*, 1994; van der Gaast *et al*, 1994; Takada *et al*, 1995; Lai *et al*, 1996; Brechot *et al*, 1997; Nisman *et al*, 1998). In addition to lung cancer, CYFRA 21-1 has been reported to be a useful marker for cervical carcinoma (Gaarenstroom *et al*, 1995; Doweck *et al*, 2000), oesophageal cancer (Brockmann *et al*, 2000; Kawaguchi *et al*, 2000), breast cancer (Nakata *et al*, 2000), gastric cancer (Nakata *et al*, 1996), and bladder cancer – (Sanchez-Carbayo *et al*, 1999). Little is known, however, about the clinical significance of serum CYFRA 21-1 in primary liver cancer, although Kashihara *et al* (1998) have reported marked high concentration of serum CYFRA 21-1 in four patients with severe advanced ICC. As cytokeratin 19 is abundant in ICC (Osborn *et al*, 1986; Balaton *et al*, 1988; Johnson *et al*, 1988; Moll *et al*, 1992), serum CYFRA 21-1 may be useful for diagnosing and monitoring these neoplasms. In assessing relations to histologic type and pathologic stage in primary liver cancer, we compared serum CYFRA 21-1 with three widely used tumour markers: AFP, CEA, and CA 19-9 in large number of patients with primary liver cancer.

## MATERIALS AND METHODS

### Patients

The study was performed retrospectively using consecutively obtained samples from 187 patients who underwent hepatic resection for primary liver cancer. Serum samples were collected just before surgery and were stored at −80°C until analysis. All patients were referred to the Department of Hepato-Biliary-Pancreatic Surgery at Osaka City University Hospital between 1994 and December 2001, and had histologically confirmed primary liver cancer. Their characteristics are listed in Table 1

**Table 1:** Characteristics of 187 patients with primary liver cancer and 87 controls with benign liver disease

*Primary liver cancer*	
Median age, years (range)	**63 (35–79)**
Gender (male : female)	**151 : 36**
*Histology*	
Hepatocellular carcinoma	**164**
Intrahepatic cholangiocarcinoma	**23**
*Stage*	
I	**28**
II	**92**
III	**36**
IV	**31**
*Benign liver disease*	
Median age, years (range)	**60 (22–76)**
Gender (male : female)	**64 : 23**
*Histology*	
Chronic hepatitis C	**63**
Chronic hepatitis B	**6**
Liver cirrhosis	**16**
Haemangioma	**2**

. The patient population included 164 patients with HCC and 23 with ICC; of the latter, six had c-HCC-CC. Tumour stage was defined according to the pathologic tumour-nodes-metastasis (pTNM) classification proposed by the International Union Against Cancer (Sobin and Wittekind, 1997).

Control blood samples were obtained from 87 patients with nonmalignant liver diseases (Table 1). These patients were diagnosed using clinical, radiologic, and laboratory criteria. Diagnoses of cirrhosis were confirmed by liver biopsy specimen examination. This study was conducted in accordance with the Helsinki Declaration and the guidelines of the Ethics Committee of our institution. Informed consent was obtained from each patient.

### Measurement of tumour markers

We measured CYFRA 21-1 using an electrochemiluminescent immunoassay (ECLIA). The assay, using an Elecsys 2010 analyser (Roche Diagnostics, Basel, Switzerland), is based on the ability of an electrochemically luminescent molecule, a tris(2,2′-bipyridyl)ruthenium (II) complex, to be repeatedly excited by tripropylamine. The system can be applied to both competitive and sandwich-format immunoassays. CYFRA 21-1 was recognised by two mouse monoclonal antibodies, a biotinylated monoclonal cytokeratin 19-specific antibody (Ks 19-1) and a monoclonal cytokeratin 19-specific antibody (BM 19-21), directed against two different epitopes of a fragment of cytokeratin 19. In the first incubation, Ks 19-1 and BM 19-21 labelled with a ruthenium complex were allowed to react, forming a sandwich complex. The next incubation followed addition of streptavidin-coated microparticles, so the sandwich complex could bind to the particulate solid phase via interaction of biotin and streptavidin. The reaction mixture then was aspirated into the measuring cell, where the microparticles were captured magnetically upon the surface of the electrode. Unbound reactants were removed with a phosphate-tripropylamine buffer. Application of voltage to the electrode then induced chemiluminescent emission that was measured by a photomultiplier.

For comparison, conventional tumour markers (CEA, CA 19-9, and AFP) were measured by a chemiluminescent immunoassay (CLIA).

### Statistical analysis

Data are given as the median with 25th and 75th percentiles of marker concentrations. Kruskal–Wallis one-way analysis of variance was performed initially for multiple-comparison tests. When this analysis was significant, pairs of groups were compared using the Mann–Whitney *U*-test. To compare abilities of tumour markers to distinguish patients with primary liver cancers from those with benign liver disease, receiver operator characteristic (ROC) curves, which correlate true- and false-positive rates (sensitivity and (1−specificity)), were constructed using the ROCKIT program (Metz *et al*, 1998a, 1998b). In addition, areas under the ROC curve (AUC) were calculated for each marker. The statistical significance of differences between the two AUCs also was determined.

## RESULTS

### CYFRA 21-1 distribution and histologic type of primary liver cancer

**Figure 1:**
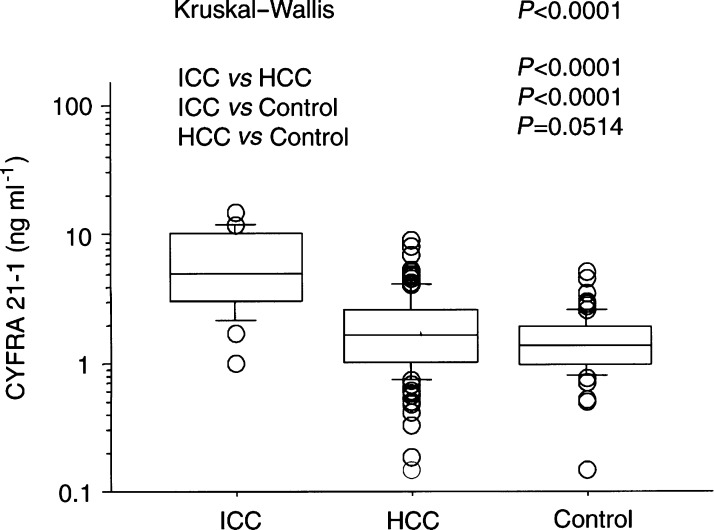
Distribution of individual serum CYFRA 21-1 concentrations in patients with primary liver cancer and benign liver diseases. Data are presented as upper and lower quartile and range (box), median value (horizontal line), and middle 90% distribution (whisker line).

Significant differences in CYFRA 21-1 concentration (median and interquartile range) were noted between patients with primary liver cancer (1.9; interquartile range 1.1–3.1 ng ml^−1^) and those with benign liver disease (1.4; 1.0–1.9; Mann–Whitney *U*-test, *P*=0.0009). Distributions of serum CYFRA 21-1 concentrations for HCC (1.7; *1.1*–*2.7*), ICC (*5.0*; 3.1–*10.7*), and benign liver disease are shown in Figure 1

. Significantly higher concentrations of CYFRA 21-1 were noted in patients with ICC than in those with benign liver disease (Mann–Whitney *U*-test, *P*<0.0001) or HCC (*P*<0.0001).

### Sensitivities of CYFRA 21-1 and other tumour markers

Table 2

**Table 2:** Sensitivity of tumour markers in patients with primary liver cancer

	**CEA (ng ml^−1^)**	**CA 19-9 (U ml^−1^)**	**AFP (ng ml^−1^)**	**CYFRA21-1 (ng ml^−1^)**
*Benign liver disease*				
Median	1.8 (1.3–2.7)	16.0 (7.0–25.8)	6.5 (3.7–11.0)	1.4 (1.0–1.9)
Cutoff value	4.8	47.0	20.3	3.0
*HCC*				
**Median**	**2.8 (1.6–3.2)**	**23.0 (12.0–44.0)**	**38.5 (8.1–328.3)**	**1.7 (1.1–2.7)**
**Sensitivity**	**5.5% (9/164)**	**33.5% (55/164)**	**59.1% (97/164)**	**17.1% (28/164)**
*ICC*				
Median	3.2 (2.4–7.1)	96.0 (30.8–862.3)	6.3 (5.1–13.8)	5.0 (3.1–10.7)
Sensitivity	34.8% (8/23)	60.9% (14/23)	17.4% (4/23)	87.0% (20/23)

Data are given as the median with 25th and 75th percentiles of marker concentrations.

Cutoff values selected according to 95% specificityin the benign group.

CEA=carcinoembryonic antigen; fetoprotein=AFP.

shows the results for serum concentration and sensitivity of AFP, CEA, CA 19-9, and CYFRA 21-1 in relation to histological type. When cutoff values were selected according to 95% specificity in the benign group, the cutoff values were 20.3 ng ml^−1^ for AFP, 4.8 ng ml^−1^ for CEA, 47.0 U ml^−1^ for CA 19-9, and 3.0 ng ml^−1^ for CYFRA 21-1. AFP demonstrated a higher sensitivity (59.1%) than other markers in patients with HCC. In patients with ICC, the most sensitive marker was CYFRA 21-1 (*87.0*%). Although CA 19-9 showed relatively high sensitivity in ICC patients (*60.9*%), the sensitivity of CA 19-9 was 33.5% in HCC patients.

### Analysis of ROC curves

**Figure 2:**
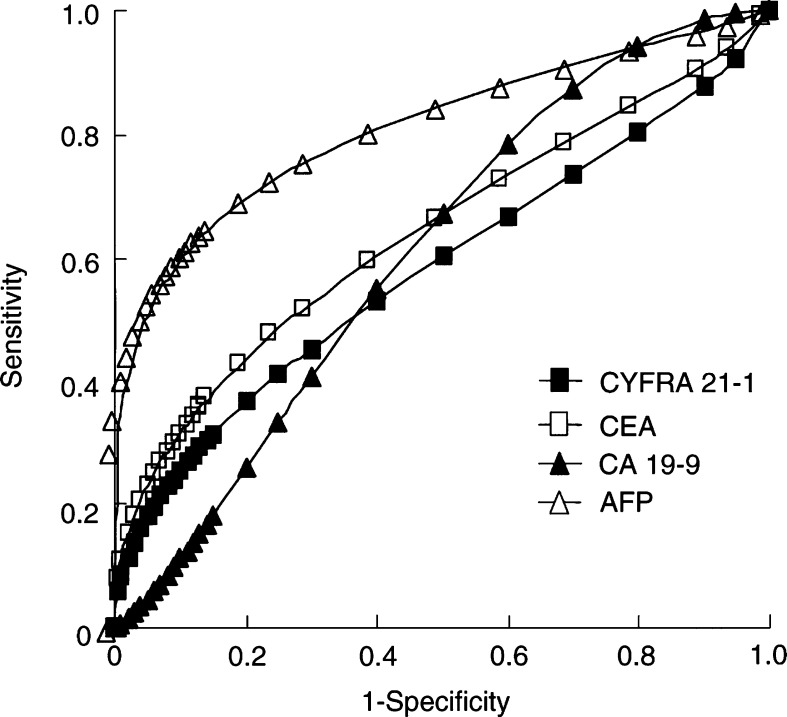
Receiver operating characteristic (ROC) curves in patients with hepatocellular carcinoma and benign liver disease were constructed.

Receiver operator characteristic curves were constructed to compare the ability of the four markers to differentiate between patients with malignant and benign liver disease. Curves for HCC (Figure 2

) and ICC (Figure 3

**Figure 3:**
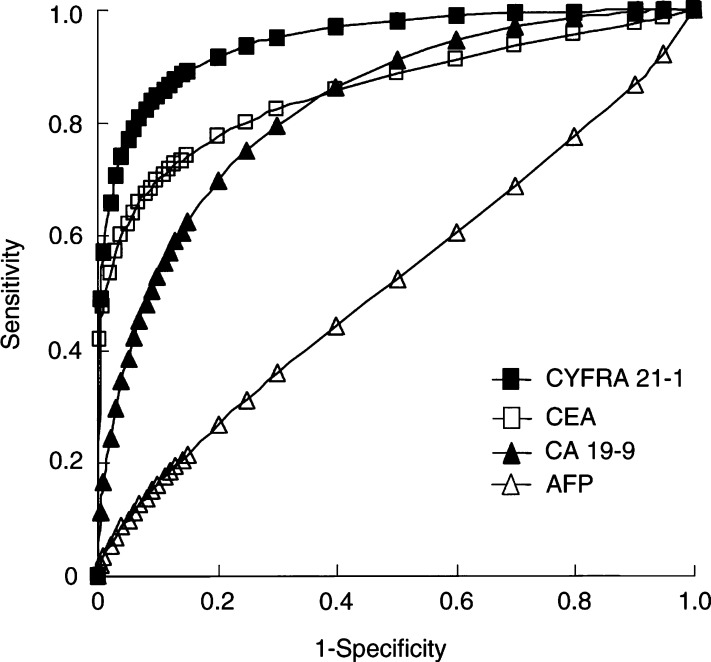
Receiver operating characteristic (ROC) curves in patients with intrahepatic cholangiocarcinoma and benign liver disease were constructed.

) in distinction to benign liver disease are illustrated. For patients with HCC, the AUC was 0.81±0.03, 0.61±0.04, 0.64±0.03, and 0.58±0.04 for AFP, CEA, CA 19-9, and CYFRA 21-1, respectively. The AUC for AFP differed significantly from that for other markers (*P*<0.0001). For patients with ICC, the AUC was 0.53±0.08, 0.81±0.05, 0.86±0.06, and 0.95±0.03 for AFP, CEA, CA 19-9, and CYFRA 21-1, respectively. A significant difference was noted between the AUC for CYFRA 21-1 and those for CEA (*P*=0.0196) and AFP (*P*<0.0001); on the other hand, no significant difference was noted between the AUC for CYFRA 21-1 and that for CA 19-9 (*P*=0.0913). In the ICC group, ROC analysis demonstrated that CYFRA 21-1 was superior to the other markers.

### CYFRA 21-1 and pathologic variables in patients with ICC

For 23 patients with ICC, CYFRA 21-1 concentrations were compared according to disease stage (Table 3

**Table 3:** Serum CYFRA 21-1 concentrations of 23 patients with intrahepatic cholangiocarcinoma

	**Number**	**Median (interquartile range)**	***P*-value**
*Tumour size (cm)*			
⩽4.0	12	3.2 (2.7–4.4)	0.0068^a^
>4.0	11	9.3 (5.0–11.6)	
*Vascular invasion*			
Present	13	8.4 (4.0–11.9)	0.0218^a^
Absent	10	2.7 (3.1–5.1)	
*Number of tumour*			
Single	12	3.6 (3.1–5.1)	0.0648^a^
Multiple	11	7.6 (3.5–12.0)	
*T factor*			
T1/2	7	3.1 (2.1–3.7)	0.0038^b^
T3	9	4.4 (3.2–6.4)	
T4	7	11.5 (7.8–12.2)	
*Lymph node metastasis*			
Present	6	6.9 (3.4–12.1)	0.3627^a^
Absent	17	4.2 (3.1–9.6)	
*Stage*			
I/II	6	3.2 (3.1–3.8)	0.0102^b^
III	10	4.4 (3.1–7.6)	
IV	7	11.5 (7.8–12.2)	

a Mann–Whitney *U*-test.

b Kruskal–Wallis test.

, Figure 4

**Figure 4:**
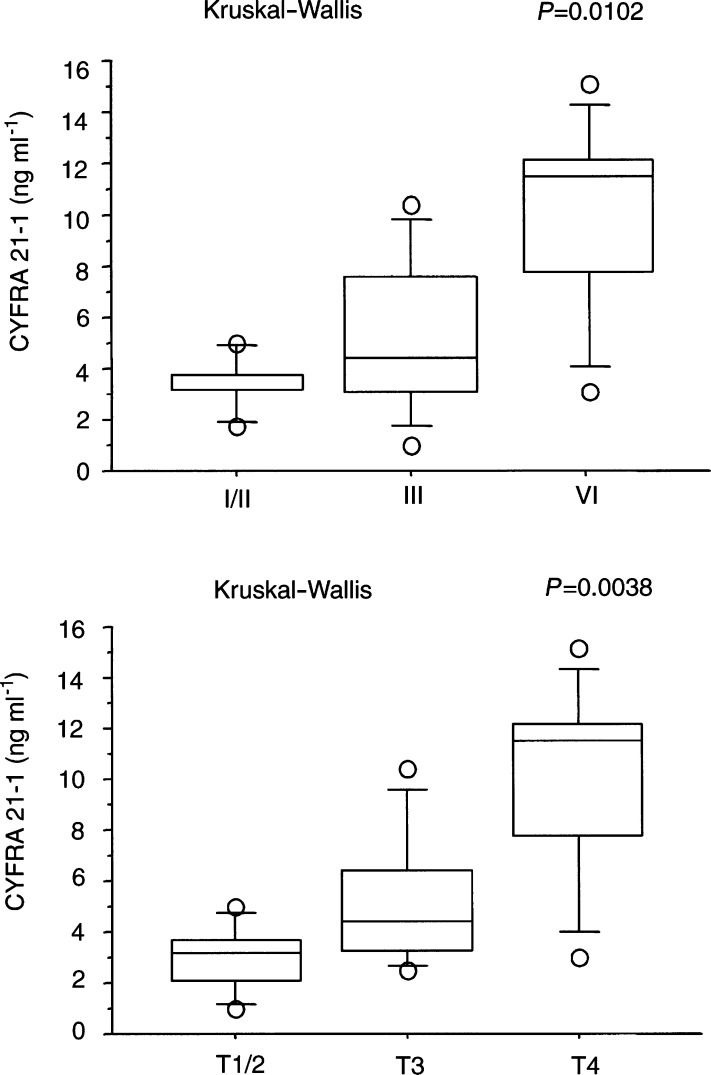
Distribution of individual serum CYFRA 21-1 values according to stage (upper) and T factor (lower) in patients with intrahepatic cholangiocarcinoma.

). Median and interquartile range of serum CYFRA 21-1 concentrations were 3.2 (3.1–3.8), 4.4 (3.1–7.6), and *11.5* (*7.8*–12.2) for stages I/II, III, and IV, respectively. An overall tendency towards an increase in serum concentration was observed from stages I/II to IV, and a significant difference was noted (Kruskal–Wallis test, *P*=0.0102). No significant difference was evident for serum CEA or CA 19-9 concentration between tumour stages (Kruskal–Wallis test; *P*=0.0774 and 0.1252, respectively). The serum CYFRA 21-1 concentration also differed significantly according to the T factor by the TNM classification (Table 3, Figure 4). Median and interquartile ranges of serum CYFRA 21-1 concentrations were 3.1 (2.1–3.7), 4.4 (3.2–6.4), and 11.5 (7.8–12.2) for T1/2, T3, and T4, respectively (Kruskal–Wallis test, *P*=0.0038). Tumour size and vascular invasion were related to serum CYFRA 21-1 concentration, while serum CYFRA 21-1 concentration did not differ according to nodal status.

## DISCUSSION

ICC, including c-HCC-CC, has been reported to carry a poorer postoperative prognosis than HCC reflecting more aggressive invasion and frequent metastasis, especially to lymph nodes (Maeda *et al*, 1995; Madariaga *et al*, 1998; Uenishi *et al*, 2000; Inoue *et al*, 2000; Uenishi *et al*, 2001). Early diagnosis and curative surgical resection therefore provide the only chance of long-term survival for patients with ICC. Despite advances and refinements, no tumour marker has shown satisfactory sensitivity or specificity for early detection of ICC, for estimating extent of disease, or for monitoring response to treatment. Thus, a reliable marker for ICC would be a valuable addition to available diagnostic tests. Cytokeratins are cytoskeletal intermediate filaments present in almost all normal and malignant epithelial cells (Moll *et al*, 1992). Characteristic combinations of cytokeratin polypeptides are expressed in different epithelia depending on the organ and/or type of differentiation (Osborn *et al*, 1986; Moll *et al*, 1992). Epithelial cells in the liver also express characteristic cytokeratins. In normal human liver, hepatocytes express cytokeratins 8 and 18, while bile duct cells also contain cytokeratins 7 and 19 (Osborn *et al*, 1986; Balaton *et al*, 1988; Johnson *et al*, 1988; Moll *et al*, 1992). Since this cytokeratin pattern ordinarily is preserved during neoplastic transformation, ICC and the CC component of c-HCC-CC contain cytokeratin 19, while HCC does not (Osborn *et al*, 1986; Balaton *et al*, 1988; Johnson *et al*, 1988; Moll *et al*, 1992). Therefore, a soluble fragment of cytokeratin 19, CYFRA 21-1, may be a useful marker for ICC. Kashihara *et al* (1998) have reported that high serum concentrations of CYFRA 21-1 in patients with large liver cancer would suggest the existence of ICC rather than HCC, since serum concentration of CYFRA 21-1 markedly elevated in four patients with ICC, compared with that in 13 patients with HCC. However, all the four patients had severe advanced and unresectable tumours. We evaluated serum CYFRA 21-1 concentrations in 23 patients with various stage ICC to determine the usefulness of CYFRA 21-1 as a marker for diagnosis of ICC, and also to identify relation between CYFRA 21-1 levels and various histological features including components of tumour staging schema.

Tumour markers such as CA 19-9 and CEA can be used in combination not only for diagnosis of gastrointestinal malignancies but also for monitoring during and in follow-up treatment. Some investigators have recommended that a possible diagnosis of ICC should be considered when a hepatic tumour is associated with high-serum CEA concentration, since serum CEA had relatively high sensitivity in patients with ICC (Kawarada and Mizumoto, 1984; Wang *et al*, 1994). Kubo *et al* (1995) reported that a high-serum CEA concentration strongly suggests ICC in patients with hepatolithiasis, even when no hepatic tumour is detected. Generally, however, serum CEA concentrations are elevated in only 20–45% of patients with ICC (Yamanaka *et al*, 1995; Chu *et al*, 1997; Harrison *et al*, 1998; Kashihara *et al*, 1998; Kim *et al*, 1999; Uenishi *et al*, 2001). In the present study, serum CEA also showed a low sensitivity for ICC (34.8%). While serum CEA may be helpful in diagnosis of some ICC and in follow-up of patients who showed elevated titres prior to surgery, it is not sufficiently sensitive or specific for reliable diagnosis of the disease (Kawarada and Muzumoto, 1984; Wang *et al*, 1994). CA 19-9 currently is in wide use, particularly for detecting bile duct cancer in patients with primary sclerosing cholangitis (PSC) (Nichols *et al*, 1993; Ramage *et al*, 1995; Bergquist *et al*, 1998; Chalasani *et al*, 2000). Serum CA 19-9 concentrations also are elevated in 65–80% of patients with ICC, and CA 19-9 has been considered the most sensitive serologic marker for diagnosis and follow-up of ICC (Kawarada and Mizumoto, 1990; Yamanaka *et al*, 1995; Kim *et al*, 1999; Uenishi *et al*, 2001). However, when we used a cutoff value giving a specificity of 95% *vs* benign liver disease, CYFRA 21-1 had the highest diagnostic sensitivity for ICC (*87.0*%). Importantly, sensitivity of serum CYFRA 21-1 for HCC was low in this study (17.1%). Although the sensitivity of CA 19-9 was relatively high in our patients with ICC (*60.9*%), its specificity for ICC was limited; serum CA 19-9 was elevated in 33.5% of HCC patients. Serum CYFRA 21-1 therefore may be a particularly useful marker for distinguishing ICC from HCC in a radiologically demonstrated hepatic tumour. However, our study could not clarify whether screening for serum CYFRA 21-1 detects very early ICC, since only two patients had a stage I tumour. Apparent sensitivity of a tumour marker in a study is influenced by several factors, particularly numbers of patients in early or advanced stages of cancer. Some investigators have indicated that serum CYFRA 21-1 may be useful for early detection of non-small-cell lung cancer, where CYFRA 21-1 elevations were common even in patients with early stage disease (Sugama *et al*, 1994; Takada *et al*, 1995). Prospective studies are needed to assess the place of CYFRA 21-1 in screening for ICC.

When two or more tests are available for use in diagnosis, comparison of ROC curves often will indicate which is best. The diagnostic test with the ROC curve enclosing the largest area is most accurate. For example, a high degree of specificity and sensitivity of CYFRA 21-1 for diagnosis of non-small cell lung cancer was shown by ROC curve findings (Pujol *et al*, 1993; Stieber *et al*, 1993; Sugama *et al*, 1994; Takada *et al*, 1995; Nisman *et al*, 1998). Analysis of the AUCs clearly showed better discrimination between ICC and benign liver diseases for CYFRA 21-1 than for CEA or CA 19-9. These results indicated that serum CYFRA 21-1 should be a useful and reliable tumour marker for ICC.

Previous studies have validated CYFRA 21-1 as a marker for monitoring disease in patients with various cancers, especially since CYFRA 21-1 concentrations and the sensitivity of CYFRA 21-1 increased progressively with clinical stage (Pujol *et al*, 1993; Sugama *et al*, 1994; Gaarenstroom *et al*, 1995; Takada *et al*, 1995; van der Gaast *et al*, 1995; Lai *et al*, 1996; Nakata *et al*, 1996; Brechot *et al*, 1997; Nisman *et al*, 1998; Brockmann *et al*, 2000; Doweck *et al*, 2000; Kawaguchi *et al*, 2000; Nakata *et al*, 2000) Our study also demonstrated that serum CYFRA 21-1 concentration was related to tumour stage of ICC, while serum CEA and CA 19-9 concentrations were not. CYFRA 21-1 concentrations differed according to the tumour size and vascular invasion, but not according to the number of tumours or nodal status. These results suggested that CYFRA 21-1 should be useful for disease staging and monitoring in patients with ICC.

In conclusion, serum CYFRA 21-1 should be a useful diagnostic test for ICC given its outstanding sensitivity. Serum CYFRA 21-1 concentrations reflected differences in ICC tumour burden, so this marker also could be applied to staging and monitoring. Prospective studies should be performed to evaluate the real impact of serum CYFRA 21-1 as a marker for ICC.
